# Experimental data on transport coefficients for developing laminar flow in isosceles triangular ducts using the naphthalene sublimation technique

**DOI:** 10.1016/j.dib.2018.03.090

**Published:** 2018-03-27

**Authors:** José Alberto Reis Parise, Francisco Eduardo Mourão Saboya

**Affiliations:** a Pontifícia Universidade Católica do Rio de Janeiro, Department of Mechanical Engineering, Rua Marquês de São Vicente, 225 – Gávea, 22453-900 Rio de Janeiro, RJ, Brazil; b Universidade Federal Fluminense, Department of Mechanical Engineering, 21410-240 Niterói, RJ, Brazil

## Abstract

The data presented in this article are related to the research article entitled "Transport coefficients for developing laminar flow in isosceles triangular ducts" (Parise and Saboya, 1999) [1]. The article describes an experiment involving the determination of transport coefficients in the laminar entrance region of 30°, 45°, 60° and 90° isosceles triangular ducts. Data were obtained by application of the naphthalene sublimation technique in conjunction with the heat to mass transfer analogy. Experimental conditions (duct sides made of naphthalene and base made of metal) simulated developing velocity and temperature fields in an isosceles triangular duct with isothermal lateral walls and adiabatic base. The Reynolds number ranged from 100 to 1800 and the duct length to hydraulic diameter ratio, from 2 to 40. The experiment consisted of mounting a test section (triangular duct) with the lateral walls made of naphthalene. Air was forced past the test section and naphthalene walls were weighed prior and after each data run, providing the rate of mass transfer for given flow conditions. Raw data, for a total of 77 experimental runs, include: test section geometry, air flow and mass transfer conditions. Processed data comprise the relevant non-dimensional groups, namely: Reynolds, non-dimensional axial duct length and Sherwood numbers.

## Specifications Table

**Table:** 

Subject area	*Engineering*
More specific subject area	*Applied Thermal Engineering*
Type of data	*Tables*
How data was acquired	*Laboratory experiment; main instrument: Sartorius precision balance; also included an air flow meter and a chronometer*
Data format	*Raw data and processed data, in tables*
Experimental factors	*Experimental runs, during which room temperature variation exceeded 0.5 K from the average temperature value, were discarded*
Experimental features	*The experiment consisted of mounting a test section (triangular duct) with the lateral walls made of naphthalene. Air was forced past the test section and naphthalene walls were weighed prior and after each data run, providing the rate of mass transfer for given flow conditions.*
Data source location	*Rio de Janeiro, Brazil*
Data accessibility	*Data are available with this article*
Related research article	*J.A.R. Parise, F.E.M. Saboya, Transport coefficients for developing laminar flow in isosceles triangular ducts*[1]

**Value of the data**

• The naphthalene sublimation technique has been applied for decades, and is still in use as attested by Refs. [2], [3], [4], [5], [6], from years 2016 to 2017. In this respect, the data here presented may provide quantitative information on issues that are not normally addressed by traditional research articles. Examples: (a) Aiming at reducing the mass transfer measuring uncertainty, how long can an experimental run last, $\Delta t_{e}$, without affecting the channel geometry? (b) What could be the maximum allowable laboratory temperature variation, $\Delta T_{lab}$?
• Access to the experimental data from this article will assist researchers and designers who have recently been working with heat and mass transfer in triangular ducts [7], [8], [9], triangular channel heat exchangers [10], [11], [12] and triangular solar air heaters [7], [13], to validate their simulation models or to benchmark their own experimental data.
• Finally, the present article may benefit works about triangular ducts with some sort of heat or mass transfer augmentation device, for example, [9], [10], [11][13], [14], [15], by providing baseline (no enhancement) data.

## 1 Data

Table 1 presents the raw data, collected over 77 experimental runs. It includes test section geometry (apex angle, duct length and triangle side length), air flow conditions (air relative humidity, mean ambient temperature, maximum departure from mean ambient temperature, air pressure at rotameter inlet, air volumetric flow rate) and mass transfer conditions (mass of sublimated naphthalene and time duration of run). Table 2, with processed data, contains: hydraulic diameter, Reynolds number, non-dimensional duct length, and the average Sherwood number based on the logarithmic mean naphthalene concentration difference.

**Table 1:** Raw data (duct geometry, air flow conditions and naphthalene sublimation).

Run	2α	L	s	ϕ	$T_{a,m}$	${\left|\Delta T\right|}_{\max}$	$P_{R}$	${\dot{V}}_{0}$	$\Delta m_{T}$	$\Delta t_{e}$
number	deg.	mm	mm	%	°C	°C	mmHg	mL/min	g	s
1	90	139.40	10.40	63	19.61	0.11	762.2	946	0.0190	7278.28
2	90	89.50	10.25	64	19.45	0.15	757.4	949	0.0218	9112.99
3	90	109.70	10.20	66	20.79	0.11	753.6	1605	0.0342	8903.81
4	90	89.50	10.25	64	19.47	0.18	756.0	1600	0.0235	8089.24
5	90	139.40	10.40	65	20.18	0.42	757.5	3550	0.0397	7200.5
6	90	89.50	10.25	64	19.39	0.31	756.6	2898	0.0304	7239.95
7	90	59.40	10.40	67	19.89	0.11	760.3	2245	0.0186	7230.82
8	90	109.80	10.30	65	19.41	0.19	759.0	4889	0.0437	7225.91
9	90	139.40	10.40	64	21.36	0.26	754.4	7832	0.0538	6242.62
10	90	59.50	10.30	64	19.62	0.23	761.0	3539	0.0256	7314.96
11	90	109.80	10.30	65	19.77	0.07	758.5	7790	0.0352	4999.71
12	90	139.40	10.40	63	18.93	0.15	758.6	11,169	0.0477	6037.60
13	90	59.50	10.30	64	19.58	0.12	758.9	7785	0.0336	7000.04
14	90	59.40	10.40	65	19.87	0.12	760.2	10,326	0.0318	5384.76
15	90	59.50	10.30	64	18.17	0.13	758.4	13,645	0.0247	4563.40
16	90	29.50	10.10	65	18.89	0.21	757.2	10,347	0.0204	5443.12
17	90	29.50	10.10	65	19.45	0.35	757.1	13,687	0.0162	4064.26
18	90	20.10	10.00	78	21.89	0.11	759.7	10,365	0.0320	7405.45
19	90	20.10	10.00	80	19.38	0.42	762.9	12,837	0.0218	7037.22
20	90	13.45	10.00	64	17.70	0.40	759.0	13,629	0.0154	5800.68
21	60	120.00	10.55	61	20.19	0.31	765.8	945	0.0183	8126.91
22	60	110.00	10.45	61	22.19	0.11	763.2	950	0.0206	7702.22
23	60	120.00	10.55	61	21.43	0.33	764.1	1597	0.0313	8403.20
24	60	119.20	10.50	61	22.45	0.15	762.8	2250	0.0339	6969.43
25	60	110.00	10.45	67	19.11	0.09	759.1	2244	0.0260	7008.32
26	60	119.40	10.40	62	20.28	0.42	764.6	3534	0.0363	6955.73
27	60	110.00	10.30	67	19.94	0.31	762.2	3537	0.0341	6044.36
28	60	60.10	10.30	66	19.10	0.40	760.7	2241	0.0199	7639.86
29	60	110.00	10.70	69	20.01	0.29	756.9	4901	0.0296	4560.23
30	60	60.10	10.30	67	19.41	0.19	758.8	3543	0.0279	7248.32
31	60	110.00	10.30	66	22.04	0.16	756.0	7833	0.0572	5116.42
32	60	30.50	10.40	70	20.12	0.28	759.3	2243	0.0174	7537.40
33	60	110.00	10.40	62	23.06	0.34	757.4	11,254	0.0592	4518.06
34	60	30.10	10.60	69	21.39	0.31	759.8	3602	0.0272	8191.33
35	60	90.10	10.40	61	22.70	0.15	758.9	11,239	0.0450	4317.92
36	60	60.30	10.35	69	17.89	0.36	760.9	7551	0.0262	6021.97
37	60	30.50	10.40	71	18.51	0.11	760.1	4878	0.0131	5345.38
38	60	60.30	10.35	70	19.45	0.40	758.1	11,182	0.0371	5429.83
39	60	30.50	10.40	62	22.21	0.19	754.1	7845	0.0233	4810.20
40	60	30.50	10.40	63	21.53	0.07	753.5	11,256	0.0235	4092.90
41	60	13.00	10.20	61	21.93	0.53	759.8	7812	0.0195	7254.12
42	60	13.90	10.50	60	23.87	0.23	754.2	13,010	0.0284	7220.87
43	45	139.70	9.90	85	24.15	0.10	765.2	952	0.0291	7330.34
44	45	109.50	9.85	85	22.19	0.41	764.9	949	0.0202	7709.24
45	45	139.80	9.90	83	23.68	0.32	764.4	1602	0.0370	7046.44
46	45	139.65	9.80	82	24.52	0.33	762.8	2258	0.0478	6897.89
47	45	139.80	9.90	82	24.40	0.05	763.4	2910	0.0548	7530.88
48	45	139.65	9.80	81	24.02	0.23	762.0	3563	0.0416	5155.91
49	45	139.65	9.80	82	22.96	0.34	763.3	4254	0.0546	6098.58
50	45	139.80	10.05	82	23.19	0.41	760.7	4905	0.0661	7016.28
51	45	139.60	10.05	82	20.23	0.22	764.5	7765	0.0587	7282.90
52	45	139.80	10.05	82	23.88	0.07	763.1	9525	0.0556	4014.85
53	45	139.60	10.05	81	21.56	0.19	761.7	11,196	0.0575	5142.09
54	45	139.80	10.05	83	21.83	0.17	762.6	12,044	0.0281	2516.16
55	45	79.60	10.10	85	22.07	0.23	766.8	6931	0.0485	6407.05
56	45	79.60	10.10	84	23.55	0.15	763.0	12,026	0.0470	4098.11
57	45	49.80	10.00	79	24.28	0.12	761.7	10,393	0.0363	4089.06
58	45	29.40	10.10	83	24.13	0.07	763.5	8674	0.0345	4855.74
59	45	30.20	10.00	81	24.45	0.10	760.8	11,257	0.0510	6804.73
60	45	20.10	10.00	81	22.75	0.25	762.1	9513	0.0219	5137.65
61	45	20.10	10.00	79	23.50	0.20	761.8	12,034	0.0276	5158.47
62	30	16.45	10.10	78	24.36	0.06	752.9	10,456	0.0326	5023.12
63	30	20.30	9.90	76	24.21	0.16	752.3	10,457	0.0396	5557.33
64	30	30.45	10.20	77	24.43	0.17	753.9	10,450	0.0260	3923.27
65	30	40.45	10.00	79	24.52	0.18	753.8	10,452	0.0569	5437.72
66	30	39.20	10.00	79	23.28	0.17	757.2	7843	0.0472	6497.25
67	30	59.30	9.90	81	22.55	0.35	758.3	9534	0.0426	5525.44
68	30	90.25	10.00	80	24.46	0.14	752.5	10,461	0.0603	4428.98
69	30	80.20	9.85	82	22.95	0.20	758.6	7813	0.0550	6774.69
70	30	118.90	10.00	84	33.69	0.21	755.8	8697	0.0480	3698.79
71	30	140.05	10.10	77	24.41	0.06	753.4	9595	0.0431	3219.95
72	30	139.90	10.10	83	22.73	0.12	759.9	7822	0.0478	4635.36
73	30	139.15	9.95	86	22.03	0.17	757.6	4916	0.0255	3141.47
74	30	100.50	10.00	86	21.57	0.23	762.4	2898	0.0202	5179.31
75	30	119.25	10.00	83	22.97	0.23	761.7	2254	0.0238	5175.61
76	30	109.80	10.00	82	23.94	0.26	753.5	1615	0.0346	7447.96
77	30	139.10	9.95	82	23.36	0.14	759.2	1607	0.0316	7100.28

**Table 2:** Processed data (hydraulic diameter and non-dimensional groups).

Run	2α	$D_{h}$	$\frac{L}{D_{h}}$	Re	$x^{+}\cdot 10^{2}$	$\overset{¯}{Sh_{b}}$
number	deg.	mm	–	–	–	–
1	90	6.092	22.9	117	7.79	3.48
2	90	6.004	14.9	119	5.02	4.88
3	90	5.975	18.4	199	3.68	5.24
4	90	6.004	14.9	200	2.98	5.38
5	90	6.092	22.9	436	2.10	5.86
6	90	6.004	14.9	363	1.64	7.54
7	90	6.092	9.8	277	1.41	6.38
8	90	6.034	18.2	611	1.19	8.67
9	90	6.092	22.9	952	0.961	7.66
10	90	6.034	9.9	442	0.891	8.83
11	90	6.034	18.2	971	0.750	9.36
12	90	6.092	22.9	1388	0.659	8.92
13	90	6.034	9.9	972	0.406	11.7
14	90	6.092	9.7	1278	0.305	14.0
15	90	6.034	9.9	1271	0.229	15.1
16	90	5.916	5.0	1313	0.152	17.4
17	90	5.916	5.0	1744	0.114	19.3
18	90	5.858	3.4	1318	0.104	24.1
19	90	5.858	3.4	1663	0.0825	22.2
20	90	5.858	2.3	1777	0.0517	33.7
21	60	6.091	19.7	132	5.98	3.04
22	60	6.033	18.2	131	5.55	3.17
23	60	6.091	19.7	220	3.58	4.26
24	60	6.062	19.7	312	2.52	4.97
25	60	6.033	18.2	315	2.32	5.68
26	60	6.004	20.0	499	1.59	6.32
27	60	5.947	18.5	503	1.47	7.79
28	60	5.947	10.1	320	1.26	6.92
29	60	6.178	17.8	666	1.07	8.59
30	60	5.947	10.1	503	0.803	9.77
31	60	5.947	18.5	1093	0.677	11.9
32	6	6.004	5.1	315	0.645	10.6
33	60	6.004	18.3	1550	0.473	12.2
34	60	6.120	4.9	492	0.400	13.3
35	60	6.004	15.0	1554	0.386	12.1
36	60	5.976	10.1	1082	0.373	12.4
37	60	6.004	5.1	692	0.294	12.7
38	60	5.976	10.1	1582	0.255	16.4
39	60	6.004	5.1	1080	0.188	17.1
40	60	6.004	5.1	1557	0.131	21.6
41	60	5.889	2.2	1107	0.0798	22.7
42	60	6.062	2.3	1760	0.0521	25.1
43	45	5.063	27.6	149	7.40	2.89
44	45	5.037	21.7	1512	5.75	2.75
45	45	5.063	27.6	252	4.39	3.75
46	45	5.012	27.9	356	3.13	4.38
47	45	5.063	27.6	454	2.43	4.47
48	45	5.012	27.9	563	1.98	5.19
49	45	5.012	27.9	677	1.64	6.33
50	45	5.140	27.2	761	1.43	6.40
51	45	5.140	27.2	1230	0.883	7.13
52	45	5.140	27.2	1472	0.739	8.43
53	45	5.140	27.2	1754	0.619	8.49
54	45	5.140	27.2	1887	0.576	8.18
55	45	5.165	15.4	1082	0.570	9.65
56	45	5.165	15.4	1855	0.332	12.3
57	45	5.114	9.7	1615	0.242	14.0
58	45	5.165	5.7	1333	0.171	17.0
59	45	5.114	5.9	1738	0.136	19.0
60	45	5.114	3.9	1486	0.106	19.0
61	45	5.114	3.9	1873	0.0839	22.1
62	30	4.012	4.1	1739	0.0943	23.2
63	30	3.392	5.2	1776	0.116	21.1
64	30	4.051	7.5	1773	0.175	12.8
65	30	3.972	10.2	1756	0.232	15.3
66	30	3.972	9.9	1333	0.296	12.5
67	30	3.932	15.1	1647	0.366	9.43
68	30	3.972	22.7	1763	0.515	9.19
69	30	3.912	20.5	1352	0.607	7.15
70	30	3.972	29.9	1481	0.809	8.17
71	30	4.012	34.9	1596	0.875	5.87
72	30	4.012	34.9	1325	1.05	5.45
73	30	3.952	35.2	843	1.67	4.75
74	30	3.972	25.3	500	2.02	3.28
75	30	3.972	30.0	385	3.12	2.93
76	30	3.972	27.6	271	4.08	3.03
77	30	5.952	35.2	265	5.13	2.46

## 2 Experimental design, materials, and methods

**Fig. 1:**
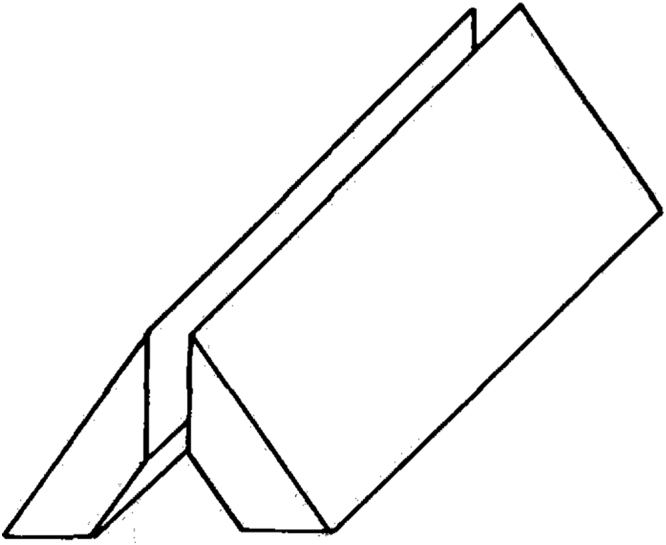
Naphthalene plates.

A simple experimental apparatus, consisting of the test section, air flow circuit and instrumentation, was constructed. Fig. 1 shows a schematic of the two naphthalene plates that form the sides of the isosceles triangular test section.

**Fig. 2:**
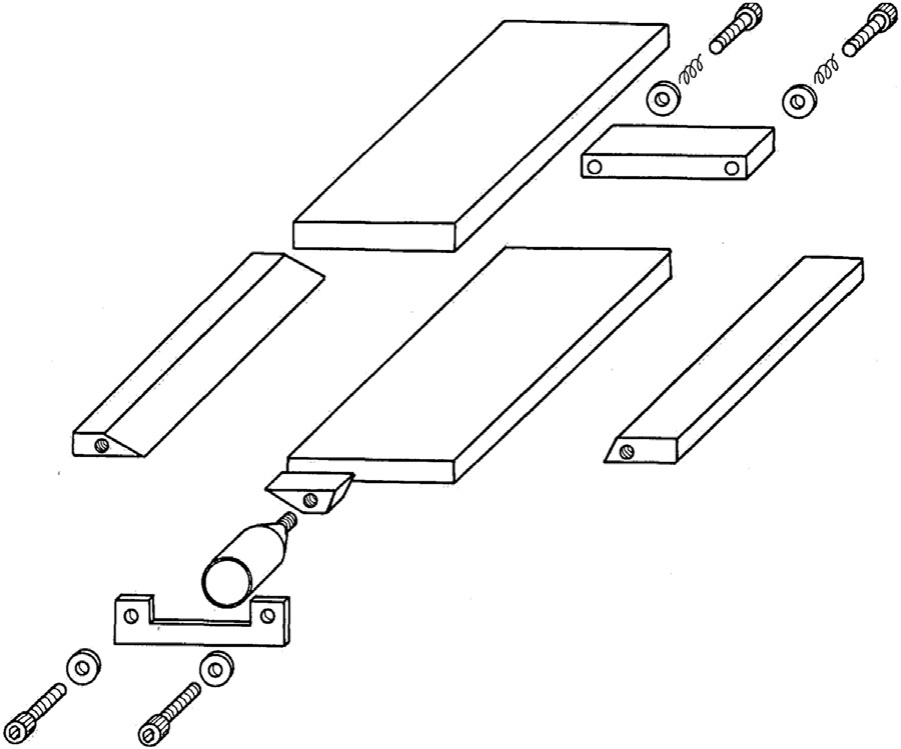
Exploded view of the casting mold of the naphthalene plates [16].

A casting technique was devised to fabricate the naphthalene plates. The casting mold comprised a number of mirror-finished brass pieces, Fig. 2, put together with the help of precision screws, guiding blocks and a pair of C-clamps.

**Fig. 3:**
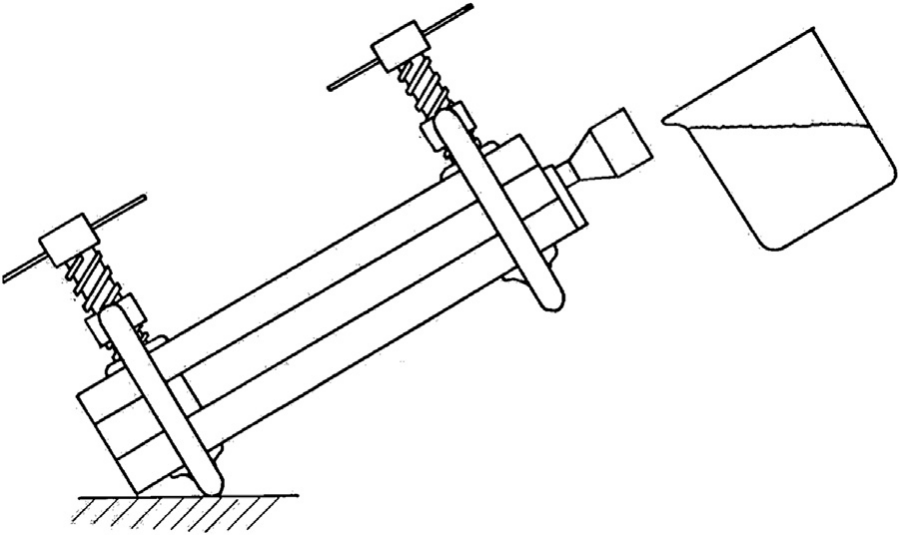
Pouring molten distilled naphthalene into the cast mold [16].

**Fig. 4:**
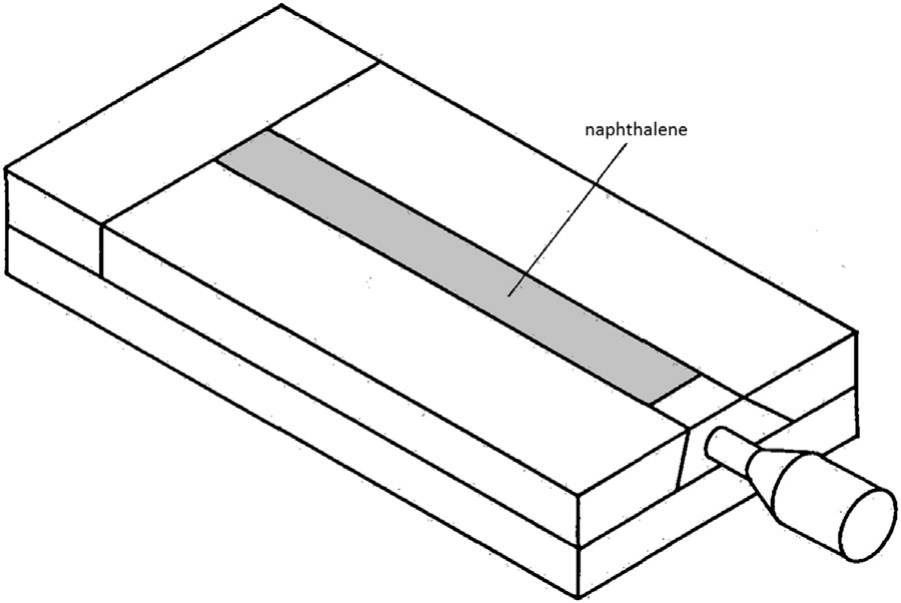
Mold with the top cover removed, exposing the naphthalene surface that will be in contact with flowing air during the experimental run [16]. (Note: solid naphthalene is originally white).

The whole set of Fig. 3 was left to cool down until the excess naphthalene left in the funnel was solidified. When removing the naphthalene plates from the mold, Fig. 4, care should be taken to avoid adhesion of naphthalene in the mold surface, as this would compromise the quality of the surface and, thence, measurements. Other surfaces of the naphthalene plate that would not participate of the sublimation process were covered with a fine adhesive tape. Provision was also made to assure that both plates were in thermal and hygroscopic equilibrium (for no less than 24 h) with laboratory ambient conditions by the start of an experimental run.

**Fig. 5:**
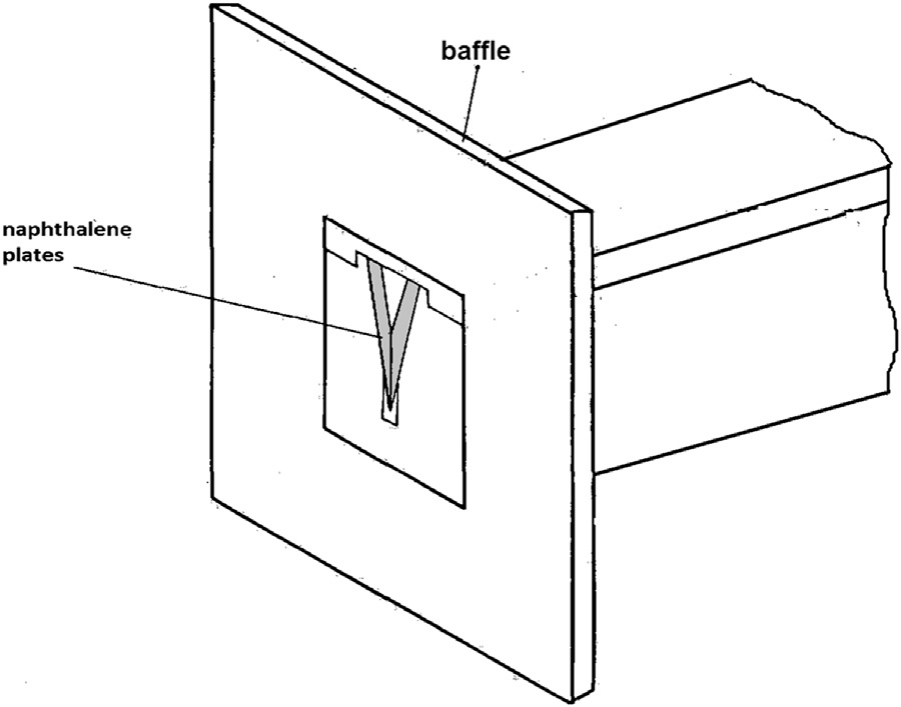
Test section and baffle (not to scale).

**Fig. 6:**
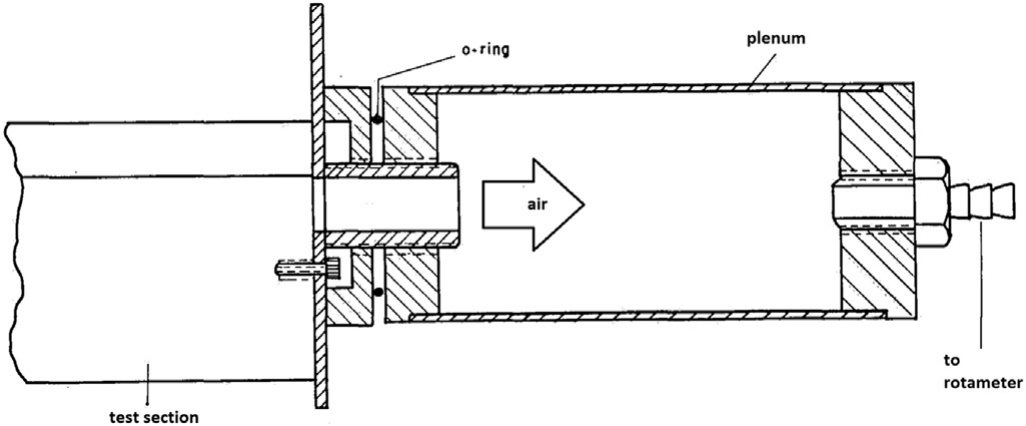
Plenum chamber downstream the test section.

The air flow circuit comprised the test section, Fig. 5, plenum chamber, Fig. 6, flow meter, flow control valves and blower. Air was drawn from laboratory into the test section and forced out to external ambient, so that inlet naphthalene concentration could always be assumed as zero. A baffle, Fig. 5, with dimensions about 30 times that of the hydraulic diameter, was placed flush with the test section frontal plane, thus contributing to an undisturbed velocity profile at the duct entrance.

The test section was further isolated from any disturbance with the installation, downstream, of a plenum chamber, Fig. 6.

**Fig. 7:**
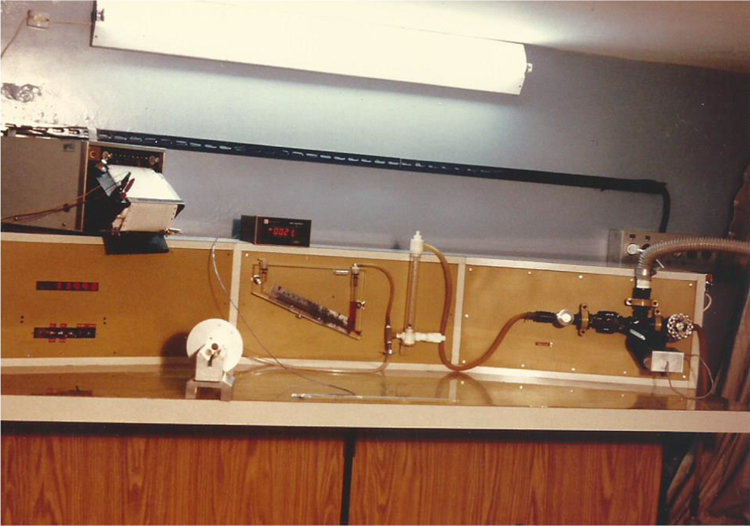
Overall picture of the experimental apparatus. Top: temperature recorder; Bottom, from left to right: chronometer, test section (with no naphthalene plates on it), inclined manometer, rotameter and set of valves for air flow rate control.

**Fig. 8:**
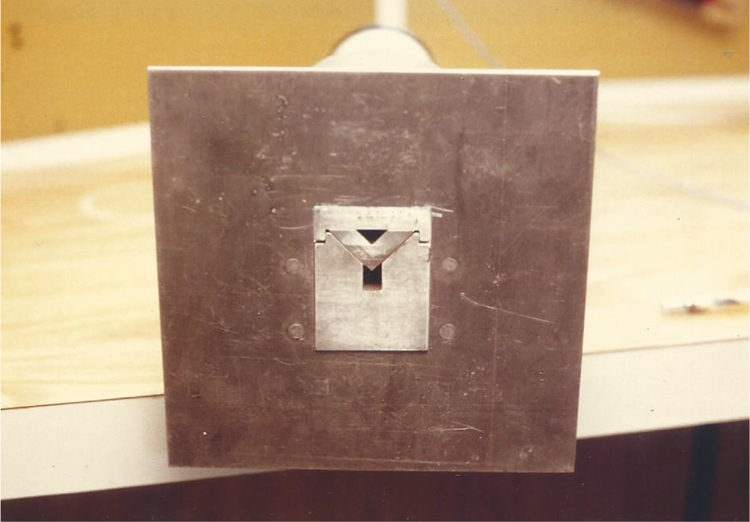
Front view of the test section with baffle and naphthalene plates installed.

Finally, Fig. 7, Fig. 8 show photographs of the experimental apparatus and a front view of the test section, respectively.

A brief description of the data reduction equations, from Table 1, Table 2, is presented next. Contrarily to the variables from raw data, SI units [m, s, K, Pa] are assumed in the following equations.

The hydraulic diameter, $D_{h}$, is given by:

(1) $$D_{h}=\frac{s\;\sin\left(2\alpha \right)}{\left(1\;+\;\sin\;\alpha \right)}$$

where 2α is the triangle apex angle and s, the length of the equal sides of the isosceles triangular duct cross section.

The Reynolds number, Re, is:

(2) Re=U0Dhν

(3) $$U_{0}=\frac{{\dot{V}}_{0}}{\frac{s^{2}\;\sin\left(2\alpha \right)}{2}}$$

where $U_{0}$ is the average air velocity across the duct, ν, the air kinematic viscosity and, ${\dot{V}}_{0}$, the air volumetric flow rate, all calculated for air at test section entrance conditions.

The non-dimensional longitudinal length, $x^{+}$, is defined as:

(4) x+=LDhReSc

where L is the longitudinal length of the test section and Sc, the Schmidt number. For naphthalene, one has:

(5) Sc=2.5

The average Sherwood number, ${\overset{¯}{Sh}}_{b}$, based on the logarithmic mean naphthalene vapor concentration difference, $\overset{¯}{\Delta \rho_{n}}$, is calculated in terms of the average mass transfer coefficient, ${\overset{¯}{\lambda }}_{b}$, the mass diffusivity of the air-naphthalene system, $D_{m}$, and the rate of total mass transfer per unit area^, $\frac{1}{A_{n}}\frac{\Delta m_{T}}{\Delta t_{e}}$.

(6) $${\overset{¯}{Sh}}_{b}=\frac{{\overset{¯}{\lambda }}_{b}\;D_{h}}{D_{m}}$$

where

(7) $${\overset{¯}{\lambda }}_{b}\;=\;\frac{1}{A_{n}}\frac{\Delta m_{T}}{\Delta t_{e}}\frac{1}{\overset{¯}{\Delta \rho_{n}}}$$

(8) $$A_{n}=2\;s\;L$$

(9) $$D_{m}=\frac{\nu }{Sc}$$

(10) $$\Delta \rho_{n}=\frac{\left(\rho_{n,w}\;-\;\rho_{n,0}\right)\;-\;(\rho_{n,w}\;-\;\rho_{n,L})}{\ln\left[\frac{\left(\rho_{n,w}\;-\;\rho_{n,0}\right)}{\left(\rho_{n,w}\;-\;\rho_{n,L}\right)}\right]}$$

where $\rho_{n,0}$, $\rho_{n,w}$ and $\rho_{n,L}$ are naphthalene vapor concentrations at the duct entrance, duct wall and duct exit, respectively, and are given by:

(11) $$\rho_{n,0}=0\;\left(air\;free\;of\;naphthalene\right)$$

(12) $$\rho_{n,w}=\frac{P_{nw}}{R_{n}\;T_{w}}$$

(13) $$\log_{10}P_{n,w}=13.564\;-\;\frac{3729.4}{T_{w}}$$

(14) $$\rho_{n,L}=\frac{1}{{\overset{˙}{V}}_{0}}\frac{\Delta m_{T}}{\Delta t_{e}}$$

## Funding sources

This work is part of the Master thesis of J.A.R. Parise. It was partially supported by CAPES, Brazilian Ministry of Education, (Post graduate program code: 31005012012P1), and by CNPq, Brazilian Ministry of Science, Technology, Innovation and Communication (Grant no. 312189/2015-0).
