# Supplementary material for: Experimental data on transport coefficients for developing laminar flow in isosceles triangular ducts using the naphthalene sublimation technique
Source: Data Brief. 2018 Mar 27;18:1350–9. doi: 10.1016/j.dib.2018.03.090 (PMC5996406; doi:10.1016/j.dib.2018.03.090)
Supplement: Supplementary file 1 — Supplementary material [file mmc1.docx]

**Conflict of Interest Statement**

Rio de Janeiro, 10^th^ March, 2018

**Manuscript title**: Experimental data on transport coefficients for developing laminar flow in isosceles triangular ducts using the naphthalene sublimation technique

**Submission number:** DIB-D-18-00264R2

The author whose name is listed immediately below certifies that he has NO affiliation with or involvement in any organization or entity with any financial interest (such as honoraria; educational grants; participation in speakers’ bureaus; membership, employment, consultancies, stock ownership, or other equity interest; and expert testimony or patent-licensing arrangements), or non-financial interest (such as personal or professional relationships, affiliations, knowledge or beliefs) in the subject matter or materials discussed in this manuscript.


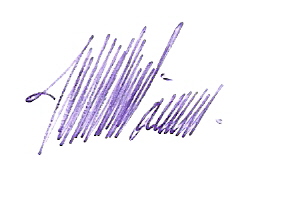
15^th^ March, 2018

Author name: José Alberto Reis Parise

The author whose name is listed immediately below is deceased.

Author name: Francisco Eduardo Mourão Saboya
